# Experiments suggesting extra-digestive effects of enteral pancreatic amylase and its peptides on glucose homeostasis in a pig model

**DOI:** 10.1038/s41598-017-07387-2

**Published:** 2017-08-17

**Authors:** Stefan G. Pierzynowski, Kateryna Goncharova, Peter C. Gregory, Björn Weström, Sergiy E. Podpryatov, Sergii S. Podpriatov, Jarosław Woliński, Hlib Repich, Nils Wierup, Liudmyla Lozinska

**Affiliations:** 1 Department of Biology, Lund University, 22362 Lund, Sweden; 2 Innovation Centre - STB, 83110 Tczew, Poland; 3 Anara, 23132 Trelleborg, Sweden; 4 Department of Biology, Institute Rural Medicine, 20722 Lublin, Poland; 5 Department of Animal Physiology, The Kielanowski Institute of Animal Physiology and Nutrition, Polish Academy of Sciences, 05110 Jabłonna, Poland; 6 Abbott Laboratories GmbH, 30173 Hannover, Germany; 7 Clinical Research Centre of Bonding/Welding Surgery and New Surgical Technologies at Kyiv Municipal Hospital Clinic No. 1, 02091 Kyiv, Ukraine; 8 Department of Clinical Sciences, Lund University Diabetes Centre, 22100 Malmö, Sweden; 9 Department of Biochemistry and Biotechnology, Vassyl Stefanyk Precarpathian National University, 76018 Ivano-Frankivsk, Ukraine

## Abstract

The studies presented were designed to highlight the impact of pancreatic enzymes on glycemic control and insulin response. Blood glucose and plasma insulin levels were monitored after intravenous, oral or direct gut glucose tolerance tests (GTT) in 6 pigs with an intact gastrointestinal tract and in 12 pigs following duodenal-jejunal bypass (DJB) surgery. In the intact pigs, pancreatic enzymes (Creon®) given orally 1 h prior to the GTT, lowered the blood glucose levels during the oral and meal GTT and reduced the plasma insulin response during the intravenous and meal GTT. In DJB pigs, blood glucose and plasma insulin levels were higher following glucose loading into the by-passed biliopancreatic limb as compared to that following glucose loading orally or into the common intestinal limb. Infusion of amylase or amylase peptides together with glucose into the biliopancreatic limb lowered blood glucose levels in DJB pigs. These preliminary data suggest new, extra-digestive, actions of enteral pancreatic enzymes – probably amylase or its peptides – on glucose homeostasis, with an reduction in net glucose absorption into the blood and in insulin response. This ability of digestive enzymes (amylase) to reduce post-prandial hyperglycaemia in an insulin-independent manner could aid in preventing the development of obesity and diabetes.

## Introduction

The biological rational for the coexistence of the endocrine and exocrine parts of the pancreas in one organ is to a large extent currently not known and the study of these parts of the pancreas has generally been undertaken separately; the endocrine pancreas by endocrinologists/diabetologists whereas the exocrine pancreas by gastroenterologists. However, over the past 40 years a number of studies have reported evidence that insulin is directly involved in the regulation of exocrine pancreatic function^1–7^. In particular, insulin appears to be important for the regulation of amylase production^8, 9^ and secretion^1, 2^. Indeed, young obese mice exhibiting hyperinsulinemia also exhibit hyperproduction of pancreatic amylase^10^, although in aged obese mice the hyperinsulinemia resulted in a lowered level of pancreatic amylase^10^ and impairment of amylase-gene expression^11^. Important for the function of such a regulatory system is the presence of a local blood flow system whereby efferent blood flows directly from the pancreatic islets to the acini, the “insuloacinar portal system”, while paracrine influences are obvious^12–14^. Further evidence for an islet-acinar axis is that experimentally induced diabetes causes a reduction in pancreatic amylase synthesis in rats^15^ and of exocrine pancreatic secretion in sheep^4^, and both type 1 and type 2 diabetes patients frequently also develop exocrine pancreatic insufficiency^16^.

Most cystic fibrosis patients have been found to suffer from diabetes, in addition to exocrine pancreatic insufficiency^17, 18^, while patients with chronic pancreatitis or pancreatic cancer also commonly develop diabetes^19, 20^; thus suggesting acinar-islet communication. This could simply be a result of the primary disease causing cellular damage that spreads to adjacent tissues and eventually results in diabetes. Furthermore, experimentally induced exocrine pancreatic insufficiency (EPI) in piglets resulted in impaired glucose utilization and insulin response to a glucose challenge, while oral supplementation with pancreatic enzymes improved blood glucose elimination^21^. Low serum levels of amylase were found to be associated with an increased risk of insulin resistance^22^ and diabetes^23^ while high salivary amylase activity was associated with better glycemic homeostasis after starch ingestion^24^. Enteral administration of amylase was also found to exert a direct influence on glucose disposal in pigs^25^. Moreover, not only do EPI piglets display arrested growth without pancreatic enzyme therapy^26^, but they also do not grow when being fed parenterally, unless provided with enteral pancreatic enzymes^27–29^. This suggests that pancreatic enzymes must have some other functions in addition to their involvement in digestion. In recent years evidence has started to appear that amylase, the main target for insulin action in intrapancreatic regulation axis, may in turn have an influence on insulin and glucose homeostasis.

The remarkable effects of bariatric surgery in improving postprandial glucose levels and eliminating type-2 diabetes in obese patients also suggests there is a gut-driven mechanism regulating islet function^30^. Intestinal bypass alone has been shown to have a weight loss independent effect on improving glucose homeostasis in rats^31, 32^. From a physiological point of view some of the most effective types of gastrointestinal bypass surgery limit the availability of pancreatic enzymes for food digestion in alimentary (AL) and common (CL) limbs^33^. Pancreatic enzymes in the so-called biliopancreatic limb (BL), which is by-passed during duodenal-jejunum bypass (DJB) surgery, are most likely partially auto-digested because of the absence of food in the limb. This, and the delayed mixing of the remaining enzymes with the food explains why the result of successful bariatric surgery has been compared to a status of functional exocrine pancreas insufficiency, characterized by the malabsorption of nutrients^34^.

The pig is commonly accepted as an appropriate model for gastro-intestinal function in humans^35, 36^. Due to similarities in morphology and physiology of the gastrointestinal systems, enzymatic and hormonal factors, ingesta transit times and digestive efficiencies bariatric surgery performed in pigs could serve as an appropriate model^35, 36^ for studying the relationship between pancreatic enzymes, gut mucosa, and glucose absorption.

**Figure 1:**
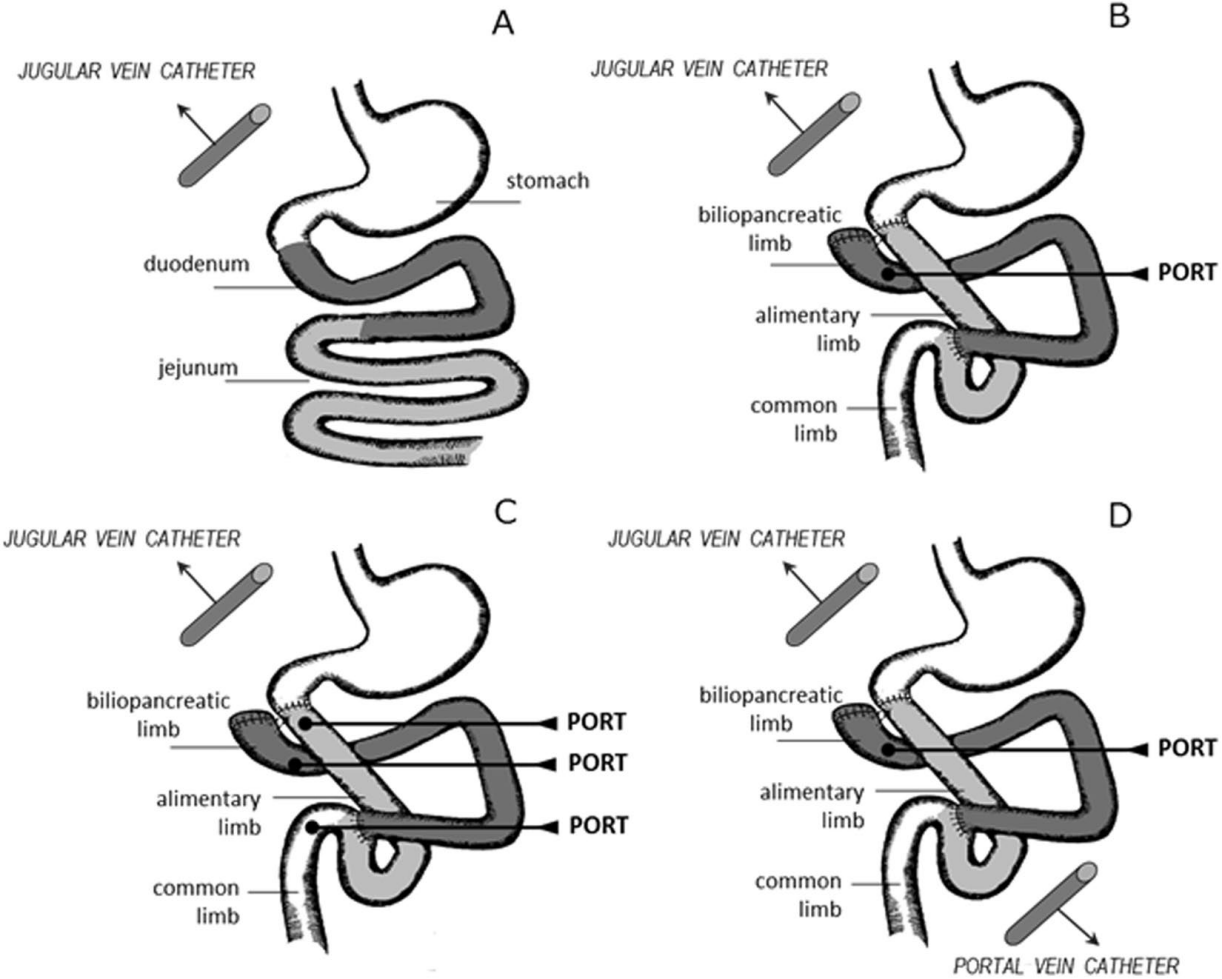
(**A**–**D**) Schematic diagrams illustrating the positions of implanted blood catheters and intestinal ports in healthy intact pigs (**A**) and in pigs following duodenal-jejunal bypass (DJB) surgery (**B**,**C**,**D**) creating a by-passed bilio-pancreatic limb (BL), an alimentary limb (AL) and a common intestinal limb (CL).

The aim of the studies detailed below was to investigate whether pancreatic enzymes, in particular amylase, have more than just digestive consequences for glucose homeostasis. Experiments involving glucose tolerance tests performed in both intact and DJB pigs (Fig. 1A–D) were designed to investigate a possible extra-digestive role of pancreatic enzymes in glucose homeostasis and insulin secretion.

## Results

### Experiment I - Effect of oral enzyme pre-treatment on glucose tolerance tests in intact pigs

#### Intravenous glucose tolerance test

**Figure 2:**
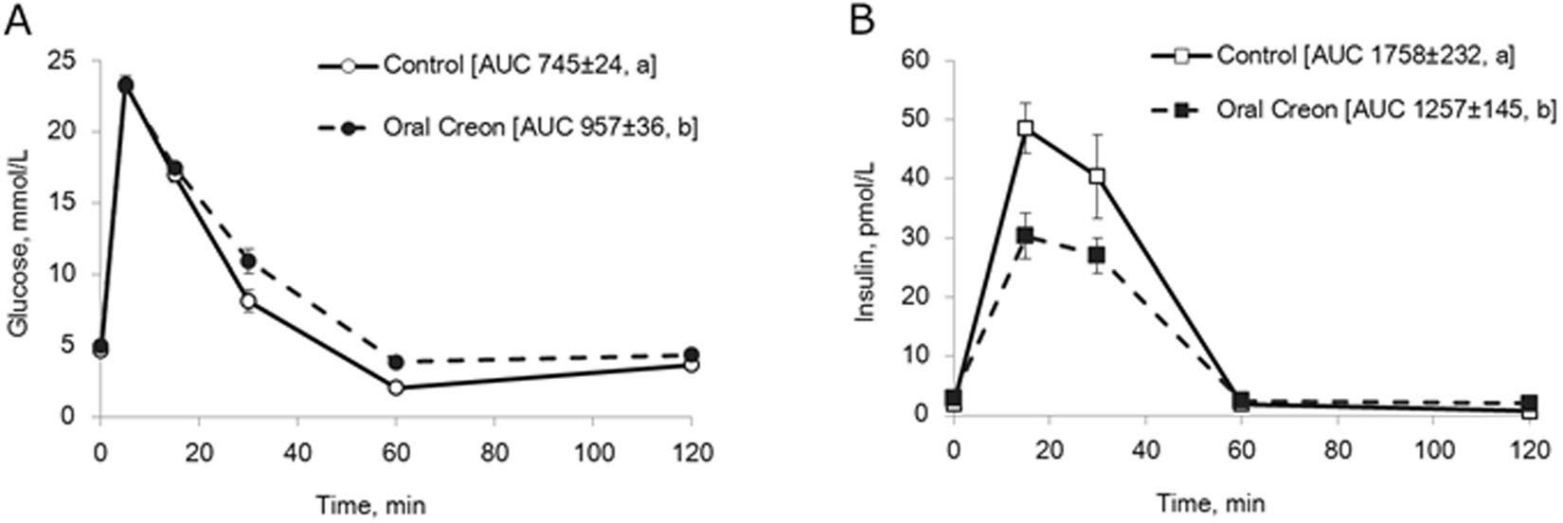
(**A**,**B**) Blood glucose (**A**) and plasma insulin concentrations (**B**) during an i.v. GTT without (control) and 1 h after oral enzyme (Creon^®^) treatment in healthy intact pigs. Data shown as mean ± SEM (n = 6) with area under the curve (AUC) values shown in brackets. Different letters indicate statistically significant differences (p < 0.05) between control and enzyme treatment.

Blood glucose levels in intact pigs (Fig. 1A) during intravenous (i.v.) GTT increased from 5 to 24 mmol/L, following i.v. glucose infusion, after which a rapid decrease in blood glucose levels were observed. The reduction in blood glucose levels occurred more slowly after oral enzyme pre-treatment than during the control test without enzyme treatment (Fig. 2A). This resulted in a higher AUC for glucose (p = 0.02) after enzyme pre-treatment. In addition, plasma insulin concentrations were significantly lower at 15 minutes following the i.v. glucose infusion after enzyme treatment as compared to that observed during the control test without enzyme pre-treatment (Fig. 2B). The AUC for insulin was significantly lower after oral enzyme treatment compared to that for the control test (p = 0.03).

#### Oral glucose tolerance test

**Figure 3:**
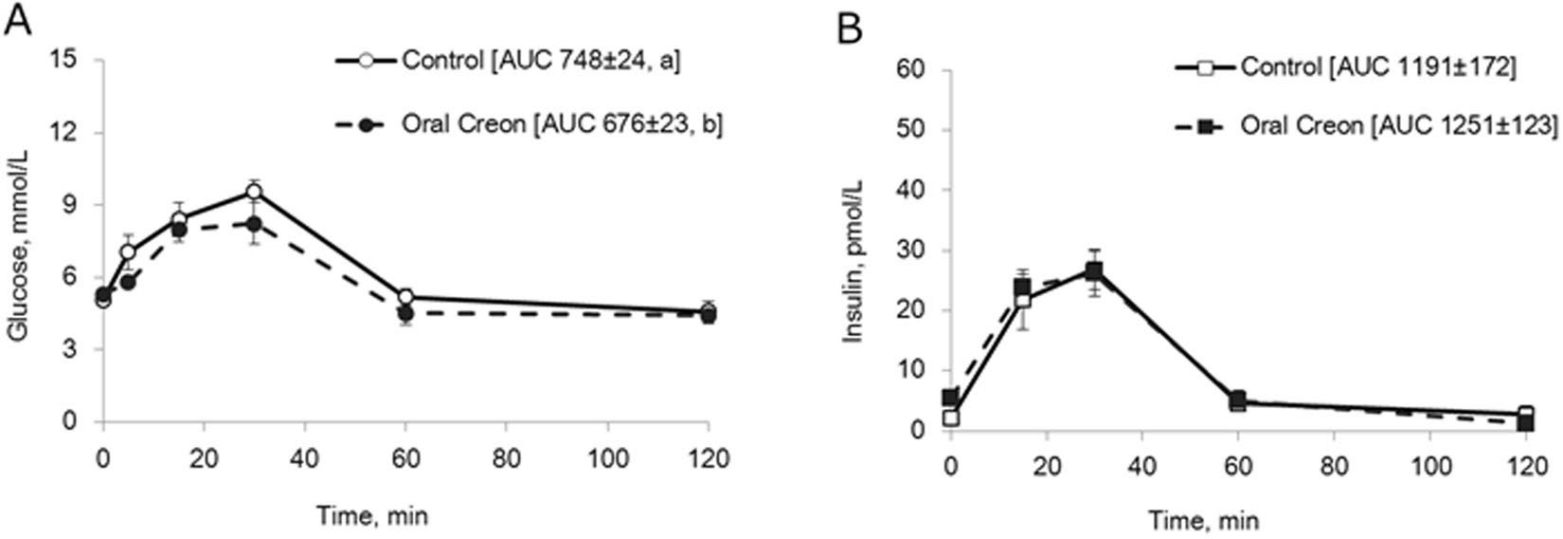
(**A**,**B**) Blood glucose (**A**) and plasma insulin concentrations (**B**) during an oral GTT without (control) and 1 h after oral enzyme (Creon^®^) treatment in healthy intact pigs. Data shown as mean ± SEM (n = 6) with AUC values shown in brackets. Different letters indicate statistically significant differences (p < 0.05) between control and enzyme treatment.

Blood glucose levels in both the control and enzyme treatment tests increased during the first 30 minutes following the oral glucose load (Fig. 3A), but with a lower calculated AUC after enzyme pre-treatment compared to the control test without enzyme pre-treatment (p = 0.04). In contrast, plasma insulin levels and the insulin AUC were similar in both the enzyme treatment and control tests (Fig. 3B).

#### Meal tolerance test

**Figure 4:**
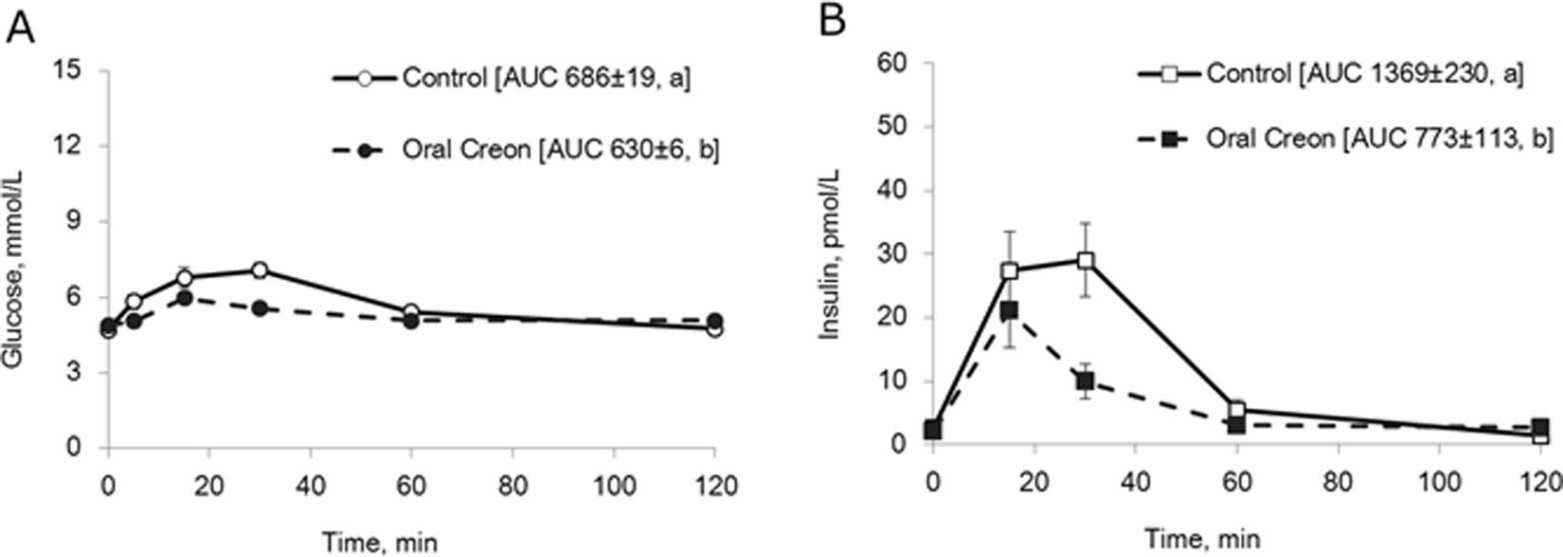
(**A**,**B**) Blood glucose (**A**) and plasma insulin concentrations (**B**) during a meal GTT without (control) and 1 h after oral enzyme (Creon^®^) treatment in healthy intact pigs. Data shown as mean ± SEM (n = 6) with AUC values shown in brackets. Different letters indicate statistically significant differences (p < 0.05) between control and enzyme treatment.

Blood glucose concentrations increased during the first 30 min following the test meal, while enzyme pre-treatment resulted in a peak at 15 min after the meal (Fig. 4A). The AUC for glucose following enzyme pre-treatment was significantly lower (p = 0.04) than that obtained during the control test without enzyme pre-treatment. Plasma insulin levels increased after the meal, reaching a plateau between 15 and 30 min after the meal during the control test. Following enzyme supplementation, plasma insulin reached a peak at 15 min and then declined (Fig. 4B). The AUC for insulin following enzyme pre-treatment was lower (p = 0.05) than that observed during the control test.

### Experiment II - Glucose tolerance tests in different intestinal limbs (channels) in DJB-model pigs

**Figure 5:**
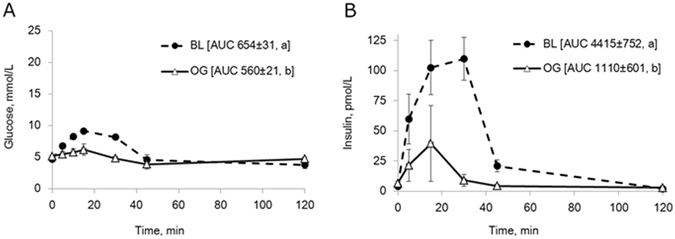
(**A**,**B**) Blood glucose (**A**) and plasma insulin (**B**) concentrations during GTT, infusing glucose directly into the biliopancreatic limb (BL), in comparison to an oral glucose challenge (OG), in DJB pigs. Data shown as mean ± SEM (n = 4) with AUC values shown in brackets. Different letters indicate statistically significant differences (p < 0.05) between control and BL-infusion.

In a first set of experiments, glucose was infused directly into the separated biliopancreatic limb (BL, Fig. 1B) which resulted in a marked increase in blood glucose and plasma insulin levels while the oral glucose tolerance test revealed only slight increases in blood glucose or plasma insulin levels (Fig. 5A,B). This was reflected in the higher AUC for both glucose (p = 0.02) and insulin (p = 0.01) after the BL glucose challenge compared to the oral glucose administation.

**Figure 6:**
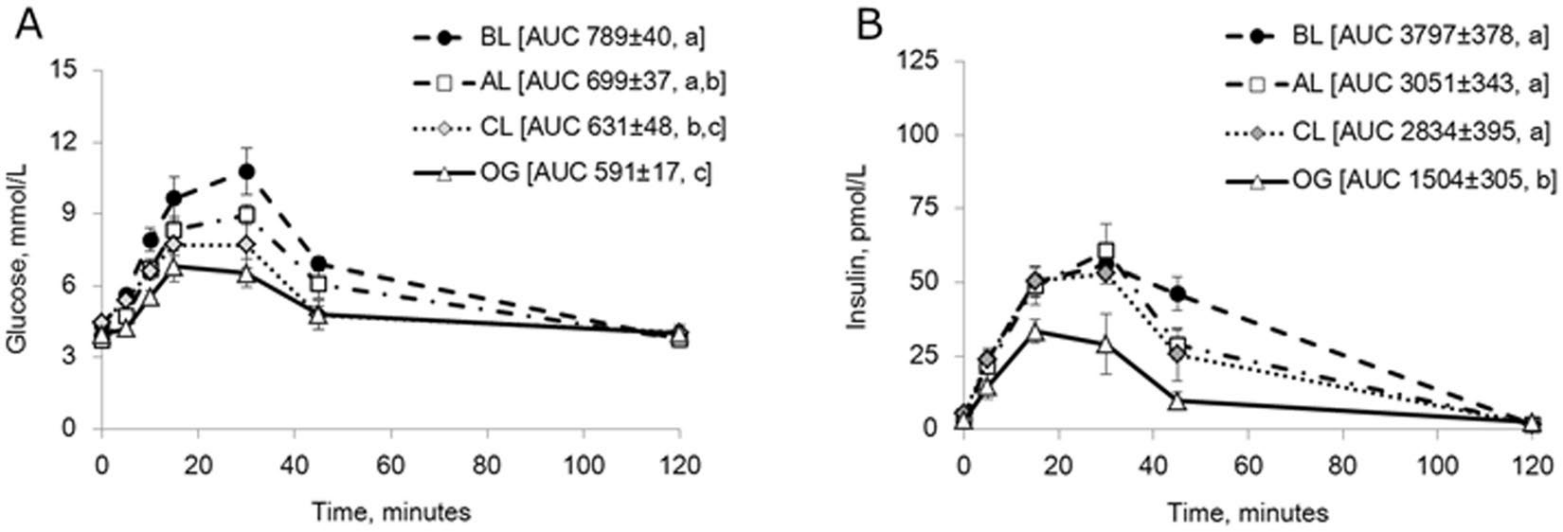
(**A**,**B**) Blood glucose (**A**) and plasma insulin (**B**) concentrations during GTT, infusing glucose either into the biliopancreatic limb (BL), the alimentary limb (AL), or the common intestinal limb (CL), in comparison to an oral glucose challenge (OG) in DJB pigs. Data shown as mean ± SEM (n = 5) with AUC values shown in brackets. Different letters indicate statistically significant differences (p < 0.05) between control and glucose infusion to the different limbs.

These results were confirmed and expanded in a second set of DJB pigs where glucose was infused into the by-passed BL limb as well as into the alimentary limb (AL) and the common (CL) intestinal limb (Fig. 1C). Infusion of glucose into the BL limb resulted in a greater increase in blood glucose level during 15–30 minutes (Fig. 6A) and larger AUC for glucose compared to that observed after glucose loading to the AL (p = 0.1) or CL (p = 0.0005), or after the oral glucose tolerance test (p < 0.0001). The plasma insulin response mirrored the glucose levels observed, and glucose loading to the BL resulted in the highest AUC for insulin, although this was not significantly different from the AUC for insulin obtained after glucose infusion to the AL or CL (Fig. 6B). The lowest insulin release was observed after oral glucose gavage.

### Experiment III – Effect of amylase supplementation on glucose tolerance in DJB-model pigs

**Figure 7:**
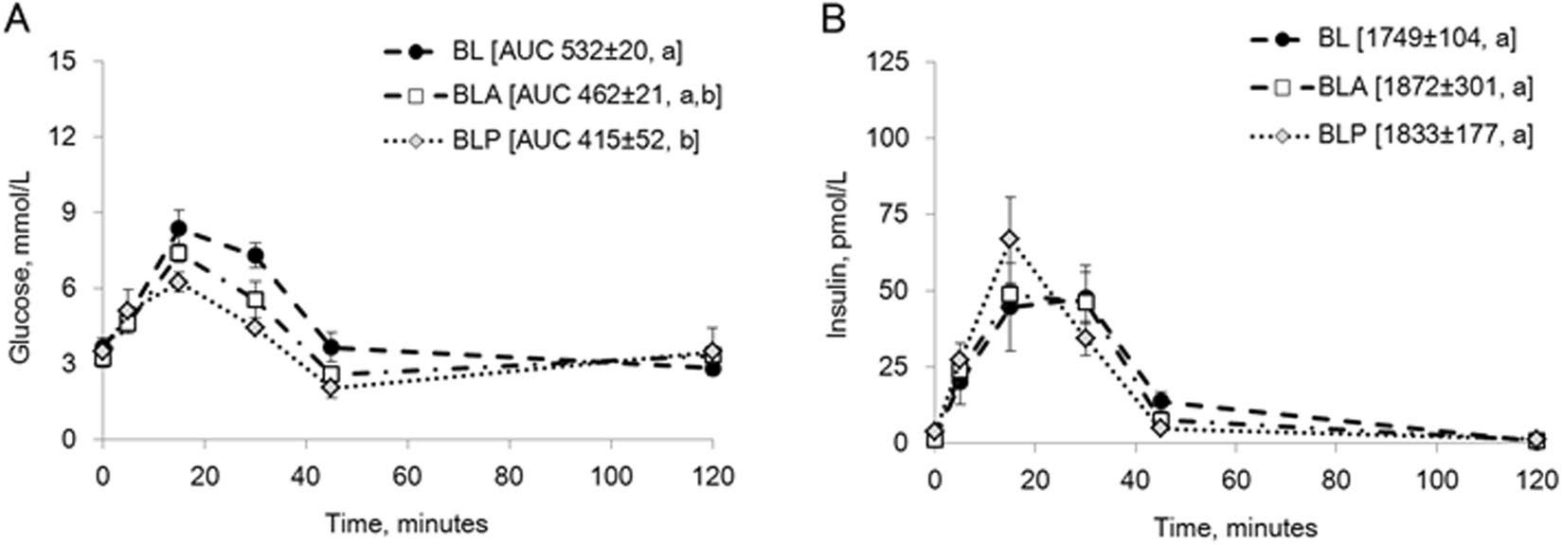
(**A**,**B**) Blood glucose (**A**) and plasma insulin (**B**) levels (in jugular vein samples) during GTT, infusing glucose to the biliopancreatic limb alone (BL), or together with amylase (BLA), or with amylase derived-peptides (BLP) in DJB pigs. Data shown as mean ± SEM (n = 3) with AUC values shown in brackets. Different letters indicate statistically significant differences (p < 0.05) between glucose infusion without (BL) and with amylase (BLA) or amylase peptide (BLP) additions.

**Figure 8:**
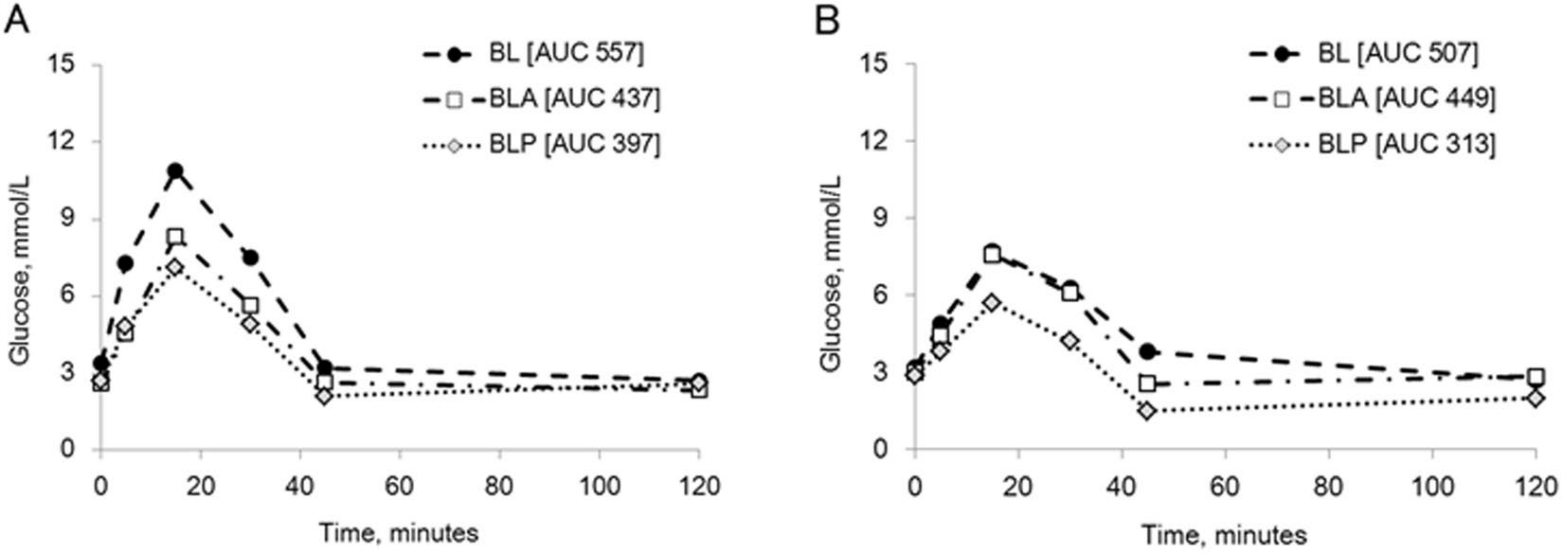
(**A**,**B**) Blood glucose concentrations in parallel blood samples taken from the portal (visceral) vein (**A**) and jugular (peripheral) vein (**B**) during the GTT from one of DJB pigs presented in Fig. 7.

Infusion of amylase (BLA) or amylase-derived peptides (BLP) 1 h before and together with the glucose solution to the BL limb (Fig. 1D) resulted in less glucose appearing in the blood (Fig. 7A), compared to that observed when glucose was infused alone (BL) (AUC glucose BL vs. BLA, p = 0.2; AUC glucose BL vs. BLP, p = 0.02), while no significant effect was seen on plasma insulin levels (Fig. 7B). In addition, parallel blood samples collected from the jugular and potal veins from one of the DJB pigs, showed that the amylase/amylase peptide lowering effects on glucose levels seen in the peripheral blood (jugular) (Fig. 8B) were evident directly after intestinal absorption in the visceral portal blood (Fig. 8A).

## Discussion

The major finding of the experiments presented is that oral pre-treatment with porcine pancreatic enzymes (Creon^®^) reduced plasma glucose levels, and the AUC for glucose, after an oral glucose load (Fig. 3A) as well as after a test meal (Fig. 4A). In contrast, slower elimination and increased glucose AUC was seen after Creon^®^ pre-treatment prior to an intravenous glucose challenge (Fig. 2A). This strongly suggests that pancreatic enzymes, when given orally, reduce net intestinal glucose absorption into the blood and reduce the speed of blood glucose elimination in intact healthy pigs due to reduced insulin release (Fig. 2B). Furthermore our results suggest, that amylase appears to be the key enzyme responsible for these effects, since glucose absorption was reduced when pancreatic amylase or amylase-peptides were given into the intestinal BL limb in DJB model pigs (Fig. 7A).

Our study adds to the accumulating evidence and suggests that amylase may be an important factor in the regulation of glucose metabolism and consequently insulin secretion (Figs 2B and 4B) and thus may have an impact on obesity development and onset of diabetes. The key role of amylase is emphasized by that amylase is produced and secreted by both the salivary and the pancreatic glands and that it is relatively resistant to digestion^37^. It has previously been described that high salivary amylase activity is associated with improved glycaemic homeostasis^24^. Conversely, it has been shown that low serum amylase is associated with the development of diabetes^23^, and with an increased risk for the development of metabolic syndrome^38^. Moreover, an association between low serum amylase and decreased plasma insulin and the development of insulin resistance have been observed^22^. Recently a positive association between low serum amylase and abdominal fat was found in older people^39^. In addition, a low copy number of the salivary amylase gene is associated with obesity^40, 41^, while a high gene copy number was suggested to be an anti-obesity factor in Mexican children^42^. The findings in our study may offer a possible explanation for these observations in that individuals with a high amylase production regulate glucose assimilation in an insulin-independent manner, which in the broader sense affects lipid metabolism (obesity), prevents insulin resistance, and reduces inflammation^41^.

Intestinal absorption of glucose mainly involves the glucose transporters, SGLT1 on the luminal side^43^ and GLUT2 on the basolateral side of the enterocytes^44^. It was recently reported that pancreatic α-amylase is able to bind to the N-glycans glycoprotein in the duodenal epithelial brush border and at high concentrations this binding inhibits the transport of glucose by SGLT1^45^. Our results may suggest an alternative overlooked mechanism for the extra-digestive actions of amylase. Amylase and/or amylase-derived products may serve as a signal to enhance the intestinal metabolism of glucose^46, 47^ thus, lowering the overall absorption of glucose into the circulation (Fig. 7A). If less glucose is transferred to the systemic circulation, glucose is presumably partly metabolised in the enterocytes during passage through the gut mucosa (first-pass effect). The difference in glucose levels due to presence of amylase and its peptides or not in the BL limb observed already in the visceral blood sampled from the portal vein as well as that sampled from the jugular vein (peripheral) in the DJB pig supports this suggestion (Fig. 8A,B).

Studies in rats with intestinal bypass highlighted an important role for the proximal small intestine, as compared to the lower part of the intestines, in the control of glucose absorption and homeostasis^31, 32^. Our studies in the DJB pigs with surgically created intestinal limbs confirmed this observation (Figs 5A and 6A). Glucose challenge directly into the biliopancreatic limb (BL), resulted in higher glucose absorption and AUC for glucose than that observed following an oral glucose challenge or a glucose challenge into the alimentary or common intestinal limbs. These results are compatible with the suggestion that the amount of glucose absorbed and the consequent secretion of insulin varies according to the different expression of the various glucose transporters in different intestinal segments^48^. On the other hand, results obtained following bypass surgery in humans and in a rat model report an increased number of the non-intestinal glucose transporter GLUT1 on the basolateral membrane of enterocytes in the alimentary limb, which increase the uptake and utilization of circulating glucose in the intestinal epithelial cells^49, 50^. Our findings introduce complementary explanations to this and suggest that duodenal amylase or its peptides infused into the by-passed BL limb, induces signalling which results in enhanced uptake and metabolism of glucose in enterocytes (Fig. 7A).

In order to better understand the mechanism behind the improved glucose metabolism in bariatric patients, one should also consider the feedback mechanisms involved in regulating pancreatic enzyme secretion. Due to DJB surgery there is lack of pancreatic enzymes in the alimentary and possible also in the common limb, producing feed-back signals increasing pancreatic enzyme secretion, among others amylase, enhancing the glucose metabolism in the intestinal mucosa. In addition, in the AL and CL limbs in bariatric patients as well as in our pig model, the lower glucose net absorption (Fig. 6A) can be dependent on salivary amylase in these limbs

Another finding from the present study was that oral administration of the pancreatic enzyme mixture (Creon^®^) in healthy growing pigs reduces the plasma insulin response (Fig. 2B) to an i.v. glucose challenge with a parallel decrease in glucose elimination (Fig. 2A). This extends previous findings from our lab in which supplementation with pancreatic enzymes was shown to lower the insulin response, as well as improve glucose disposal during an i.v. GTT in exocrine pancreas insufficient pigs^21^. We have also shown that the administration of oral bacterial amylase caused a reduction in insulin response to an i.v. GTT in healthy growing pigs^25^. The inhibitory effect on plasma insulin levels suggests that pancreatic enzymes, most likely amylase and/or its peptides, could be primarily described as prandial “decretin”– like factors which act from the gut lumen (Figs 3B and 6B) to regulate the amount of circulating insulin^51^. However, it remains to be determined whether insulin inhibition results indirectly due to increased degradation by the liver or directly through reduced beta-cell production. This has to be high-lighted by testing the effect of enteral pancreatic enzymes on the circulating C-peptide/insulin levels in the future. Factors which decrease blood glucose concentrations following ingestion of a meal, independently of insulin, might help postpone insulin desensitization and slow the development of obesity and delay the onset of type-2 diabetes. The regulation/initiation of insulin-independent glucose disposal by amylase and amylase-derived peptides could protect pancreatic beta cells from overstimulation and eventual exhaustion and cell death, which usually occurs within years of insulin overproduction.

In summary, the present study suggests extra-digestive enteral actions of pancreatic enzymes, specifically amylase and its derivatives, on blood glucose homeostasis. Firstly, enhancing intestinal glucose metabolism, thus lowering net glucose absorption and secondly, lowering insulin secretion, thus acting as a “decretin” during the prandial phase.

## Materials and Methods

### Animals

The experiments were conducted on 6 healthy pigs with an intact gastrointestinal tract (GIT) (intact pigs) and on 12 pigs that underwent duodenal-jejunal bypass (DJB) surgery. At the start of the study the pigs, females and castrates, (Swedish Landrace × Yorkshire × Hampshire) had a body weight of between 12–30 kg. For the duration of the study the pigs were housed in separate pens (1.0 × 1.5 m), equipped with a water dispenser, sawdust for bedding material and a heating lamp. The pens had windows to allow social interaction between neighbouring pigs. The study was approved by the Malmö/Lund Ethical Review Committee on Animal Experiments and all experiments were performed in accordance with relevant guidelines and regulations.

The pigs were fed dry standard feed (Växtill 320, Lantmännen, Sweden; 50% starch, 3.5% fat, 17.5% protein + free amino acids) corresponding to 4% of their body weight daily, half the ration was given in the morning, between 08:00–09:00, and the other half in the afternoon, between 16:00–17:00. If glucose loading experiments were planned for the next day, the afternoon meal was given earlier at 15:00 to ensure a stable fasting state (about 18 hours) prior to the glucose challenge. The pigs had free access to water for the duration of the experiments.

### Surgery

All pigs underwent surgery to insert an intravenous (i.v.) catheter into the jugular vein (Fig. 1A). Then 12 pigs also underwent DJB surgery with the creation of: i) an intestinal port just to the biliopancreatic limb (Fig. 1B); ii) intestinal ports to the biliopancreatic (ca 100 cm long), alimentary (ca 100 cm long), and to the remaining common limbs (Fig. 1C); iii) intestinal ports to the biliopancreatic limb and an additional portal vein catheter (Fig. 1D). All ports were inserted into the proximal (oral) part of the created intestinal limbs.

Following an overnight fast, surgery was performed on pigs after pre-medication with azaperone (Stresnil^®^, Janssen Pharmaceutica, Belgium, 2.2 mg/kg, intramuscularly). The pigs were anaesthetized using an inhalation mask, with a 0.5–1.5% air mixture of Fluothane^®^ (Astra Läkemedel, Södertälje, Sweden) in O_2_ as a carrier gas, at approximately 0.5–1 L/min, in a closed-circuit respiratory flow system (Komesaroff Medical Developments, Melbourne, Australia). Post-operative pain was prevented by administration of buprenorphine (Temgesic^®^, Schering-Plough AB, Stockholm, Sweden, 0.01 mg/kg, intramuscularly) for 1 day following surgery. Ampicillin (Doktacillin, Astra Läkemedel, Södertälje, Sweden) was administered i.v. (15 mg/kg) and at the incision site (250–500 mg) at surgery and for up to 7 days after surgery.

At the end of the experimental period, the pigs were euthanized by an i.v. injection of an overdose of pentobarbital sodium (Allfatal Vet. Omnidea, Stockholm, Sweden, 100 mg/kg,) and post-mortem examinations were performed.

### Experimental design

#### Experiment I

Previous results in our laboratory showed that orally administered pancreatic-like enzymes of microbial origin reduced insulin release during an intravenous (i.v.) glucose tolerance test (GTT) in pigs^25^. The present experiment was designed to confirm these results but using a species-matched porcine pancreatic enzyme preparation (Creon^®^ 10 000, Abbott Healthcare Products Ltd, Southampton, United Kingdom).

Six intact healthy pigs, with an initial body weight of approximately 20 kg, were fitted with a jugular vein catheter for use in the study (Fig. 1A). Six GTT experiments were performed on each pig, first under control conditions and then after oral pretreatment with pancreatic enzymes. For the latter (enzyme pre-treatment), 1 hour prior to glucose loading, the contents of 4 capsules of Creon^®^ were given to the pigs by means of a syringe in the mouth during a period of 2 minutes, followed by 20 ml of tap water. The experiments were performed every second day in the following order: i) i.v. GTT, ii) oral GTT, iii) meal GTT, iv) i.v. GTT after enzyme administration, v) oral GTT after enzyme administration, vi) meal GTT after enzyme administration. For the i.v. GTT, glucose was administered as a 50% solution (1 g glucose/kg bwt) via the *jugular vein* catheter, which immediately thereafter was flushed with 10 ml of 0.9% sterile saline solution. For the oral GTT, the same solution of glucose was given orally into the mouth within 1 minute using a syringe. For the meal GTT the pigs were fed 2.0 g of the commercial feed/kg bwt within approximately 1 minute. The dose of glucose (as dietary starch) was calculated to be similar (1 g of glucose/kg bwt) for each type of GTT.

#### Experiment II

In these experiments, we aimed to investigate glucose absorption and insulin plasma levels after glucose infusion to the different intestinal limbs (channels) that had been created by the duodenal-jejunal bypass (DJB) surgery and in which, the presence of endogenous pancreatic enzymes is likely modified.

In the first set of experiments, 6 weeks after the DJB surgery and implantation of a port to the biliopancreatic limb (Fig. 1B), 4 pigs with a body weight of approximately 52 kg were used. The pigs were tested with a glucose load to the biliopancreatic limb (BL) and the response was compared to that observed following an oral GTT with 1 g glucose/1 kg bwt (OG).

In the second set of experiments, 5 pigs were subjected to DJB surgery and implantation of ports to each of the 3 intestinal limbs created (Fig. 1C). Two weeks later, the DJB pigs were subjected to four GGTs. Glucose (1 g glucose/kg bwt) was loaded every second day into the different intestinal limbs, biliopancreatic (BL), common (CL) or alimentary limbs (AL), and finally orally (OG) as described in experiment 1.

#### Experiment III

This experiment was designed to test the possible effects of pancreatic amylase or its ‘proteolysis-derived’ peptides on glucose absorption and insulin release.

Three pigs, with a body weight of about 18 kg, underwent DJB surgery with simultaneous implantation of an intestinal port to the biliopancreatic limb (BL) and jugular and portal vein catheters (Fig. 1D). Starting from 4 weeks after surgery, the pigs were subjected to GTTs every second day where glucose was loaded to the BL either alone, or together with porcine amylase, or amylase-derived peptides. Porcine pancreatic amylase (Type VI-B, A3176, ≥ 10 units/mg solid, Sigma-Aldrich, St. Louis, MO, USA), 2.5 g in tap water, was administered 1 h prior to GTT and 2.5 g mixed with glucose solution (1 g of glucose/kg bwt) during GTT via the port to the biliopancreatic limb. Five amylase-derived peptides, of 7–11 amino acids in length (obtained by computational digestion of amylase with porcine pancreatic proteinases), were synthesized (GL Biochem Ltd., Shanghai, China). The amylase-peptides, 0.4 g mixed in 10 mL tap water, were administrated 1 h prior to GTT, and an additional 0.4 grams of amylase-peptides were mixed with glucose solution and loaded during GTT via the port to the biliopancreatic limb.

### Blood sampling

In the GTT experiments, 6 to 7 repeated blood samples were collected before and during 120 minutes after glucose loading. Blood samples were collected via the jugular (and the portal in experiment III) vein catheter into 5 ml syringes containing of Na-EDTA (0.04 mg/ml) and a protease inhibitor (1 000 kIU/ml, Trasylol, Bayer, Leverkusen, Germany). The blood samples were immediately placed on ice before they were centrifuged at 3000 × g for 15 minutes at 4 °C, and plasma was separated and stored in at −20 °C until further analysis.

### Analyses

Blood glucose concentrations were measured directly following blood sampling using a glucometer and test strips (Accu-Chek^®^ Aviva, Roche Diagnostics, Germany).

Plasma insulin concentrations were measured using a porcine insulin ELISA kit (Mercodia, Uppsala, Sweden), mainly according to the manufacturer’s instructions. A modification was made in order to ensure that insulin in the samples was within the detectable range. Fifty μl of plasma was used instead of the recommended 25 μl and, the antibody-enzyme conjugate was reduced from 100 μl to 75 μl to maintain the total volume. Due to this modification, all insulin values presented may not be regarded as absolute values, but as relative and only comparable to values obtained using the same method.

### Statistical analysis

Data are expressed as mean ± standard error of mean (SEM). All statistical analyses were carried out using the R (v. 3.0.1) programming environment. The area under the glucose and insulin curves (AUC) were compared using a paired t-test and a mixed-effect model followed by a Tukey post hoc test when comparing more than two groups. In all statistical analyses p ≤ 0.05 was considered significantly different.

### Data Availability

The datasets generated and analysed during the current study are available from the corresponding authors on reasonable request.
